# Is the current therapeutic dosage of nadroparin adequate for neonates and infants under 8 months with thromboembolic disease? a population pharmacokinetic study from a national children’s medical center

**DOI:** 10.3389/fphar.2024.1331673

**Published:** 2024-01-31

**Authors:** Yewei Chen, Jianger Lan, Lin Zhu, Min Dong, Yi Wang, Zhiping Li

**Affiliations:** ^1^ Department of Pharmacy, National Children’s Medical Center, Children’s Hospital of Fudan University, Shanghai, China; ^2^ Division of Clinical Pharmacology, Cincinnati Children’s Hospital Medical Center, Cincinnati, OH, United States; ^3^ Department of Pediatrics, University of Cincinnati, Cincinnati, OH, United States; ^4^ Department of Neurology, National Children’s Medical Center, Children’s Hospital of Fudan University, Shanghai, China

**Keywords:** nadroparin, pharmacokinetics, neonates, infants, precision dosing

## Abstract

**Objectives:** Nadroparin, a low-molecular-weight-heparin is commonly used off-label in neonates and infants for thromboembolic events prevention. However, the recommended dosing regimen often fails to achieve therapeutic target ranges. This study aimed to develop a population pharmacokinetic (PK) model of nadroparin to determine an appropriate dosing regimen for neonates and infants less than 8 months.

**Methods:** A retrospective chart review was conducted on patients treated with nadroparin at Children’s Hospital of Fudan University between July 2021 and December 2023. A population PK model was developed using anti-Xa levels, and its predictive performance was evaluated internally. Monte Carlo simulations were performed to design an initial dosing schedule targeting anti-Xa levels between 0.5 and 1 IU/mL.

**Results:** A total of 40 neonates and infants aged less than 8 months with gestational age ranging from 25 to 41 weeks treated with nadroparin were enrolled in the study for analysis. A one-compartment PK model with first order absorption and elimination was adequately fitted to the data. Creatinine clearance was identified as a significant factor contributing to inter-individual variability in clearance. The typical population parameter estimates of clearance, distribution volume and absorption rate in this population were 0.211 L/h, 1.55 L and 0.495 h^-1^, respectively. Our findings suggest that current therapeutic doses of nadroparin (150–200 IU/kg q12 h) may result in subtherapeutic exposure, thus higher doses might be required.

**Conclusion:** The present study offers the first estimation of PK parameters for nadroparin in preterm or term neonates and infants less than 8 months utilizing the model. Our findings have potential implications for recommending initial personalized dosages, particularly among patient populations exhibiting similar characteristics.

## Introduction

Thromboembolic events (TEs) in neonates and infants have become a growing concern attributed to advancements in diagnostic techniques, treatment modalities and supportive care. Both venous and arterial thrombosis can result in significant morbidity and mortality among hospitalized infants (Schmidt and Andrew, 1995; Sirachainan et al., 2018; El-Naggar et al., 2020). Currently, there is a lack of approved anticoagulant drugs for pediatric use. Despite limited available data for pharmacokinetic (PK) studies of low-molecular-weight heparins (LMWHs) in neonates and children, there is an expanding off-label use of LMWHs based on treatment guidelines (Monagle et al., 2012). Derived from unfractionated heparin (UFH), LMWHs offer several advantages over UFH including reduced bleeding risk, convenient administration, higher bioavailability, predictable anticoagulant response, and decreased need for frequent monitoring (Dolovich et al., 2000; Klaassen et al., 2019).

Nadroparin, a commonly used LMWH, enhances the inactivation of factor II and factor Xa when it binds to endogenous anticoagulant protein antithrombin III (ATIII). Through inhibiting the activation of thrombin (factor IIa) by factor Xa, nadroparin effectively interrupts the coagulation pathway (Barradell and Buckley, 1992). Due to LMWH being a mixture of polysaccharides that includes biologically inactive species, direct measurement of LMWH levels is not feasible. When nadroparin interacts with ATIII, this complex leads to an increased plasma anti-Xa activity, which can be quantified using a chromogenic assay and is considered directly proportional to nadroparin plasma concentration. Thus, the anticoagulant effect of nadroparin is indirectly monitored by measuring anti-Xa activity (Cornelli and Fareed, 1999; Duplaga et al., 2001). Nadroparin undergoes partial degradation in the liver through depolymerization and desulphurization processes and primarily excreted *via* renal elimination (Frydman, 1996; Hirsh et al., 1998). The target therapeutic range (TTR) for nadroparin is derived from adult anti-Xa levels and recommends subcutaneous administration twice-daily with an anti-Xa level ranging from 0.50 to 1.0 IU/mL at 2–6 h post-injection (Monagle et al., 2012).

However, the extrapolation of adult findings to vulnerable neonates and infants may pose potential risks, given the ontogenic characteristics of hemostasis processes that influence both thrombosis physiopathology and the response to antithrombotic agents in neonates. Anti-Xa concentrations below TTR would be associated with an increased risk of recurrent TE, while concentrations above TTR might elevate bleeding risk (Nieuwenhuis et al., 1991; Montalescot et al., 2004). Compared to adults, children require escalating dosages of all LWMHs as they age in order to achieve TTR (Nohe et al., 1999; Klaassen et al., 2019). Neonates and infants typically necessitate higher nadroparin dosages for reaching TTR, however, caution should be exercised by physicians when increasing nadroparin dosages in this population due to increased bleeding risk. Limited data exists regarding nadroparin dosages requirements for achieving TTR in preterm and term neonates. Population PK models serve as a robust tool to assist clinicians and facilitate personalized drug therapy by incorporating patient-specific characteristics, dosing information, drug concentrations, and accounting for intra- and inter-patient variability. Despite the existing controversies surrounding optimal dosages for neonates, no population PK studies have been conducted on nadroparin in this vulnerable group. Therefore, the objective of this study is to develop a population PK model for nadroparin usage among neonates and infants under 8 months in order to determine an appropriate dosage regimen.

## Methods

### Patients and data collection

Retrospective single-center PK study was conducted at Children’s Hospital of Fudan University from July 2021 to December 2023. This study enrolled preterm or term neonates and infants under 8 months with suspected or diagnosed arterial or venous thrombosis who were receiving nadroparin (Fraxiparine^®^; GlaxoSmithKline, Brentford, UK). All eligible patients were treated in accordance with the local protocol, receiving subcutaneous nadroparin at a dose of 150–200 IU/kg q12 h. Participants with anti-Xa levels below the limit of quantitation were excluded from pharmacokinetics evaluation. The protocol was approved by the Ethics Committee of our hospital.

The following data were collected from electronic medical records: gender, gestational age (GA), postnatal age (PNA), postmenstrual age (PMA), birth body weight (BBW), body weight (BW), height (HT), body surface area (BSA, $BSA{\left(m\right)}^{2}=\sqrt{\frac{HT\left(cm\right)*BW\left(kg\right)}{3600}}$ ), alanine transaminase (ALT), aspartate transaminase (AST), total bilirubin (TBIL), direct bilirubin (DBIL), urea nitrogen (BUN), serum creatinine (SCR), creatinine clearance rate (CLCR, $CLcr\left(mL/\min/1.73m^{2}\right)=k*HT\left(cm\right)/Scr\left(mg/dL\right)$ , where k = 0.33 for preterm infants and k = 0.45 for term infants throughout the first year of life) (Schwartz et al., 1984), cystatin C (CysC), and serum albumin (ALB).

### Blood sampling and anti-Xa determination

Due to the lack of available data on nadroparin usage in pediatric patients, no specific guidelines were established for the timing of sample collection. Blood samples for analysis of anti-Xa levels were obtained 4 hours after nadroparin administration, typically within 72 h following the initial dose or any subsequent dosage adjustment, or as deemed necessary by the clinician. The samples were then placed in tubes containing 3.2% buffered sodium citrate solution with an anticoagulant to blood ratio of 0.1:0.9 (vol/vol). All samples were centrifuged at 4000 RPM for 5 min at room temperature within 1 hour after collection to obtain plasma samples. Subsequently, anti-Xa levels were measured promptly using an anti-Xa clotting assay (STA®-liquid ANTI-Xa; Diagnostica Stago, Asnières, France). The lower limit of quantification (LLOQ) was 0.1 IU/mL and the calibration curve demonstrated linearity within the range of 0.1 and 2.00 IU/mL.

### Pharmacokinetic analysis

#### Model development

Population PK analysis of nadroparin was performed using the NONMEM program (version VII, Icon Development Solutions, Ellicott City, MD, United States). The first-order conditional estimation (FOCE) method with interaction was employed throughout the model-building process. A one-compartment disposition model with first-order absorption was used to describe anti-Xa levels. The model parameters included apparent clearance (CL/F), apparent volume of distribution (V/F), and absorption rate constant (Ka). Interindividual variability, assumed to follow a log-normal distribution, was assessed by an exponential model (Eq. 1).

$$P_{i}=P_{p}\cdot \exp\left(\eta_{i}\right)$$ (1)

Where *P*_*i*_ represents the individual parameter value, *P*_*p*_ represents the population parameter estimate. *η*_*i*_ is defined as a symmetrical distributed random term with zero mean and variance omega (Sirachainan et al., 2018). Residual unexplained variability was evaluated by testing various error models including proportional error, additive error, or a combination of both (Eq. 2).

$${OBS}_{i}=IPRED\cdot \exp\left(\varepsilon_{1}\right)+\varepsilon_{2}$$ (2)

Where *OBS*_*i*_ represents the observation, *IPRED* represents the individual prediction, and *ε*_*n*_ represents the symmetrically distributed random term with zero mean and variance sigma (Sirachainan et al., 2018).

During the procedure for determining the covariates for the model, each covariate (gender, GA, PNA, PMA, BBW, BW, HT, BSA, ALT, AST, TBIL, DBIL, BUN, SCR, CLCR, CysC and ALB) that may affect the inter-individual variation was analyzed. As many weight-related covariates were highly correlated in this population, weight was *a priori* selected to be included as a descriptor in the model before confirming other weight-related covariates. Allometric scaling was used to account for the influence of body size on pharmacokinetics. Clearance was scaled to a total weight of 70 kg, using an allometric exponent of 0.75 (Anderson and Holford, 2008) (Eq. 3).

$$CL/F={CL}_{std}\cdot {\left(BW/70\right)}^{0.75}$$ (3)

Where *CL/F* represents the clearance, *CL*_*std*_ represents the clearance in an adult with a body weight of 70 kg, and BW represents the bodyweight. Postmenstrual age with sigmoid Emax maturation function was tested to explore the effect of maturational changes on clearance (Eq. 4).

$$CL/F={CL}_{p}\cdot \left(\frac{1}{1+{\left(\frac{PMA}{TM50}\right)}^{-\gamma }}\right)$$ (4)

Where *CL*_*p*_ represents the population parameter estimate, PMA represents postmenstrual age, TM_50_ represents the postmenstrual age at which clearance is 50% of that of the mature value, and *γ* represents the Hill coefficient for clearance used to determine the steepness of the sigmoid decline. Exponential models were investigated for continuous covariates (Eq. 5), while category variable equations were applied for dichotomous covariates (Eq. 6).

$$P_{i}=P_{p}\cdot {\left({Cov}_{i}/{Cov}_{Median}\right)}^{\theta }$$ (5)

$$P_{i}=P_{p}\cdot \left(1+\theta \cdot {Cov}_{i}\right)$$ (6)

Where *P*_*i*_ represents the individual parameter estimate of the *ith* subject, *P*_*p*_ represents the population parameter estimate, *Cov*_*i*_ is the covariate of the *ith* subject, *Cov*_*Median*_ represents the population median for the covariate, and *θ* is the exponent. For nested models, the selection of covariates followed a forward inclusion and backward elimination process based on comparisons of the objective function value (OFV). A reduction in OFV by 3.84 (*p* < 0.05) served as the criterion for forward inclusion, whereas stricter criteria (an increase in OFV by 6.63, *p* < 0.01) were applied for backward elimination. In cases where two or more covariates significantly improved the model fit, the covariate resulting in the greatest reduction in OFV remained within the model. For non-nested models, the Akaike information criterion (AIC), calculated using Pirana software (ver. 2.7.1; Pirana Software and Consulting BV, http://www.pirana-software.com/), was utilized to determine superior models with lower AICs.

#### Model evaluation

Models were evaluated using graphical goodness-of-fit diagnostic plots, including observed concentrations (DV) plotted against population predicted concentrations (PRED), DV plotted against individual predicted concentrations (IPRED), conditional weighted residuals (CWRES) plotted against time (TIME), and CWRES plotted against PRED.

The stability of the final model was assessed by non-parametric bootstrap (Ette et al., 2003). One thousand datasets were generated by randomly resampling from the original dataset. Each bootstrap dataset was fitted with the final population pharmacokinetic model, and all model parameters were estimated accordingly. The medians of bootstrap estimates along with their corresponding 95% confidence intervals were calculated and compared with those obtained from the original dataset. If there were no significant differences in parameter values, it could be proved that the model was stable.

A visual predictive check (VPC) was performed to evaluate the predictive performance of the model (Holford, 2005). One thousand datasets were simulated based on the final model. The observed concentration *versus* time data was graphically overlaid with the median values, as well as the 5th and 95th percentiles derived from the simulated data profiles. The precision of the model was determined by evaluating whether the observed concentration data fell within the 5th and 95th prediction interval.

#### Simulations

To evaluate the adequacy of different dosing regimens of nadroparin in achieving the prespecified target ranges for anti-Xa levels, a maximum posteriori Bayesian analysis was performed using the final model. A total of 1,000 subjects were randomly sampled with replacement from the study cohort. Empirical Bayesian estimates were obtained for model parameters associated with inter-individual variability, which were subsequently utilized to calculate individual PK parameter values. Based on these parameter values, steady-state anti-Xa levels at 4 h post-dosing were calculated for various predefined dosing regimens (150, 200, 250 and 300 IU/kg q12 h) in neonates and infants under 8 months. The proportion of simulated subject profiles achieving predicted anti-Xa levels within the range of 0.5–1 IU/mL was calculated for each dosing regimen. Considering the uncertain safety profile of nadroparin in treating thrombosis in neonates and infants, the maximum dose was limited to 300 IU/kg in this simulation.

## Results

### Study population

**FIGURE 1:**
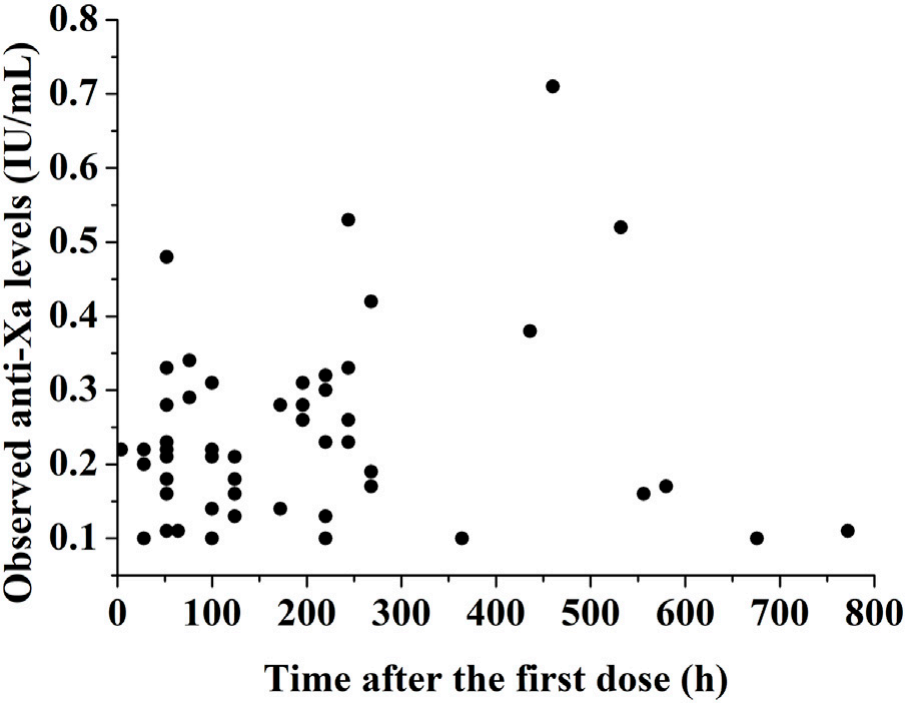
Observed profile of anti-Xa levels over time following administration.

A total of 51 preterm or term neonates and infants under 8 months treated with nadroparin were enrolled in the study. Eleven patients were excluded due to providing only samples below the lower limit of quantification. The remaining 40 patients underwent pharmacokinetic analysis and their baseline characteristics are depicted in Table 1. Figure 1 illustrates the observed profile of anti-Xa levels over time following administration.

**TABLE 1:** Baseline characteristics of patients.

Characteristic	Mean (±SD)	Median	Range
No. of patients/samplings	40/56		
Gender (Boys/Girls)	23/17		
GA (weeks)	35.2 (4.3)	36.8	25.0–41.3
<28 weeks	n = 1 (2.5%)		
28–32 weeks	n = 9 (22.5%)		
32–37 weeks	n = 10 (25%)		
≥37 weeks	n = 20 (50%)		
PNA (days)	58.4 (60.4)	40.0	3.0–224.0
PMA (weeks)	43.5 (9.5)	40.4	30.7–69.0
BBW (kg)	2.6 (1.1)	2.9	0.6–4.6
BW (kg)	3.7 (2.0)	3.1	1.2–9.3
HT (cm)	49.0 (9.9)	50.0	28.0–70.0
BSA (m^2^)	0.2 (0.1)	0.2	0.1–0.4
ALT (U/L)	61.9 (120.5)	17.7	3.2–429.3
AST (U/L)	71.8 (68.4)	41.7	16.1–277.1
TBIL (μmol/L)	53.2 (65.3)	24.6	1.8–299.6
DBIL (μmol/L)	14.6 (39.8)	7.0	0.5–250.2
BUN (mmol/L)	3.2 (2.7)	2.2	0.4–12.0
SCR (µmol/L)	42.4 (58.1)	29.7	6.7–374.8
CLCR (mL/min/1.73 m^2^)	73.8 (62.8)	51.1	5.3–314.7
CysC (mg/L)	1.2 (0.4)	1.1	0.7–3.0
ALB (g/L)	32.3 (5.0)	33.4	21.8–44.1
Locations of thrombosis			
Venous thrombosis	n = 23		
Lower limb	n = 7 (30.4%)		
External iliac vein	n = 5 (21.7%)		
Inferior vena cava	n = 4 (17.4%)		
Femoral vein	n = 2 (8.7%)		
Umbilical vein	n = 1 (4.3%)		
Portal vein	n = 2 (8.7%)		
Jugular vein	n = 2 (8.7%)		
Arterial thrombosis	n = 17		
Neonatal cerebral infarction	n = 10 (58.8%)		
Thrombosis abdominal aorta	n = 1 (5.9%)		
Arterial thromboembolism	n = 6 (35.3%)		

Note: GA, gestational age; PNA, postnatal age; PMA, postmentrual age; BBW, birth body weight; BW, body weight; HT, height; BSA, body surface area; ALT, alanine transaminase; AST, aspartate transaminase; TBIL, total bilirubin; DBIL, direct bilirubin; BUN, urea nitrogen; SCR, serum creatinine; CLCR, creatinine clearance rate; CysC, cystatin C; ALB, serum albumin.

### Population PK modeling

A one-compartment model which describes first-order absorption from a subcutaneous compartment and first-order elimination from the central compartment, effectively captured the observed data. Inter-individual variability was estimated for clearance, volume distribution and the absorption rate constant. The variance for volume distribution and the absorption rate constant were fixed to zero, as they approached zero during the model-building process. The inclusion of creatinine clearance to clearance produced the most significant decrease in the OFV and inter-individual variance of clearance. Residual variability was best described by a proportional error model. Table 2 presents parameter estimates for the final model. Structural parameters clearance, volume distribution and absorption rate constant were accurately estimated with a relative standard error below 20%. All other parameters were well-estimated with relative standard errors below 30%.

**TABLE 2:** Parameter estimates of nadroparin final model and bootstrap validation in preterm or term neonates and infants.

Parameter	Final model		Bootstrap	
	Population estimate	RSE (%)	Median	95% CI
Structural model				
CL/F (L/h)	0.211	9.4	0.207	0.063–0.259
V_d_/F (L)	1.55	13.7	1.51	0.02–2.79
k_a_ (h^-1^)	0.495	16.4	0.495	0.073–1.09
Covariate model				
CL/F_CLCR	0.238	29.2	0.235	0.056–0.381
Inter-individual variability (%CV)				
CL/F	26.5	29.6	24	0.7–41.6
Residual variability (%CV)				
Proportional residual error	35.5	10.0	34.6	27.2–41.5

Note: CL/F, clearance; V_d_/F, volume of distribution; k_a_, absorption rate constant; CLCR, creatinine clearance rate; CV, coefficient of variation of the parameter values; RSE, relative standard errors; CI, confidence interval.

### Model evaluation

**FIGURE 2:**
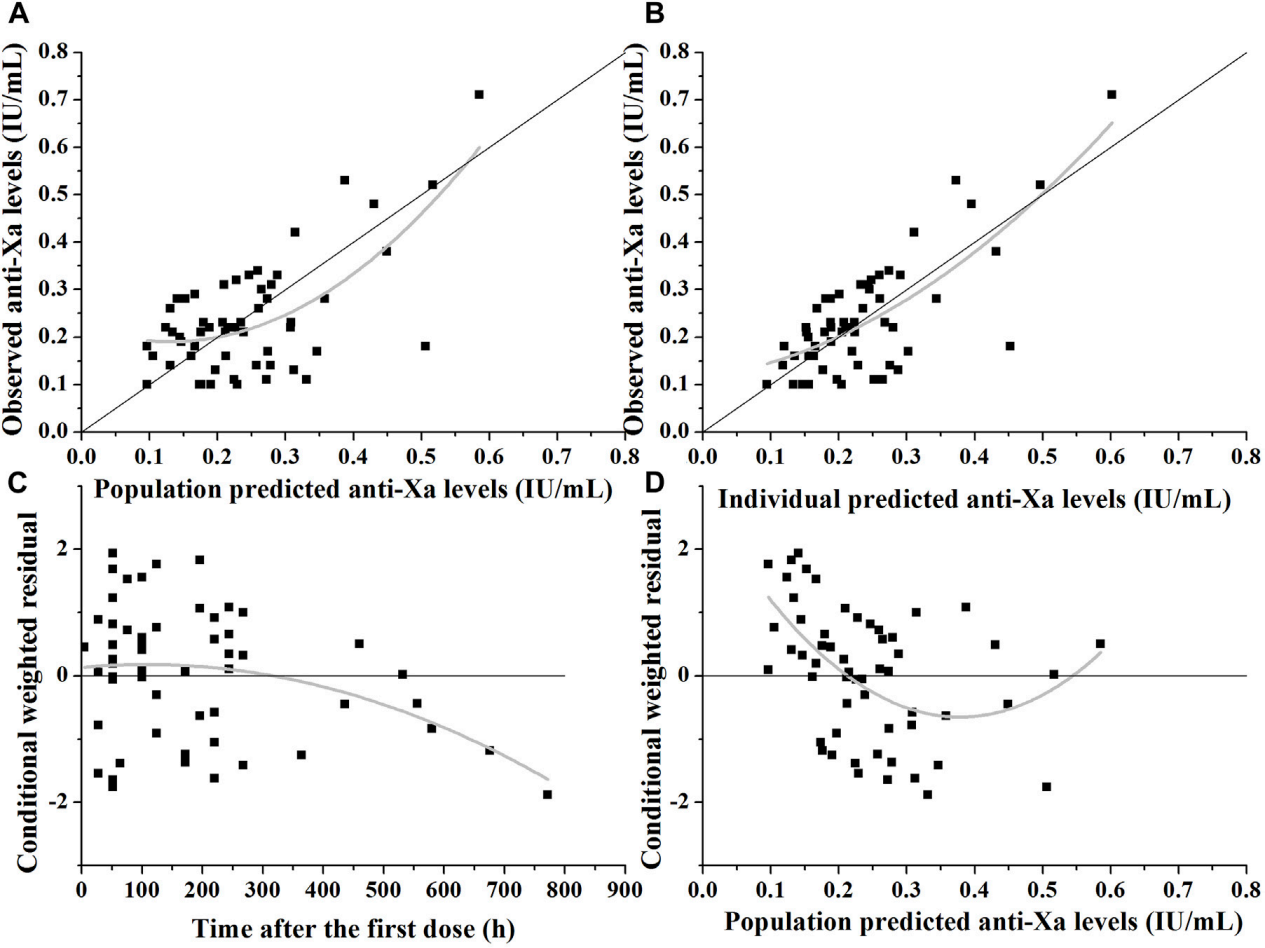
Diagnostic plots of the nadroparin final model. **(A)** The observed *versus* population-predicted anti-Xa levels. **(B)** The observed *versus* individual-predicted anti-Xa levels. **(C)** Conditional weighted residual *versus* time after dose. **(D)** Conditional weighted residual *versus* the predicted anti-Xa levels.

The goodness-of-fit plots in Figure 2 demonstrate that the model adequately predicts the anti-Xa levels. In Figure 2A, a slight deviation is observed in the population predicted anti-Xa levels, which is effectively corrected when considering the covariate creatinine clearance and inter-individual variability on CL, as depicted in Figure 2B. The predicted anti-Xa levels for the observed data exhibit a symmetrical distribution around the line of identity (y = x). Furthermore, there is no significant bias shown for the residual unexplained variability of the final model, as indicated by the symmetric display of conditional weighted residuals with time after dose or population predicted anti-Xa levels around y = 0 (Figures 2C, D). However, some bias towards lower population predicted anti-Xa concentrations can be observed in Figure 2D; despite thorough examination of these data points, unfortunately, we were unable to identify the source of this bias. Nevertheless, it is noteworthy that conditional weighted residuals with population predicted anti-Xa levels fall almost entirely within −2 and 2.

The results of one thousand bootstrap replicates for nadroparin are summarized in Table 2. Out of the total number of runs, 986 successfully converged. All medians for the parameter estimates obtained from the bootstrap procedure were similar to the values derived from the final population model. Moreover, the parameters from the bootstrap procedure followed a normal distribution and contained all parameter estimates from the final population model. The results demonstrated that the estimates for the population PK parameters were precise, indicating stability of the final model.

**FIGURE 3:**
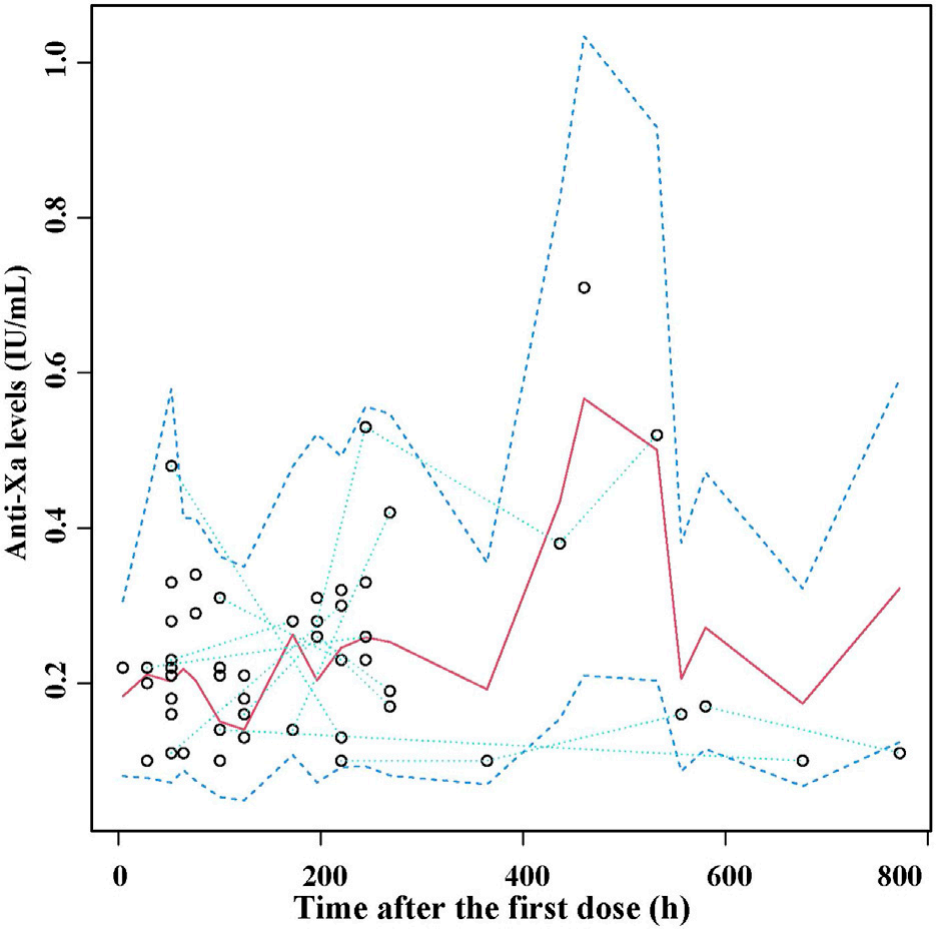
Visual predictive check (VPC) of the final nadroparin model. Circles represent the observed anti-Xa levels, upper and lower dashed lines represent 5th and 95th percentiles of simulations, median solid lines represent 50th percentile of simulations.

The VPC plot for the final model is illustrated in Figure 3. The observed median exhibited good agreement with the simulated 5th and 95th percentiles, and approximately 98% of observed data fell within the 90% prediction interval of simulation, thus indicating the good predictive performance of the final model.

### Simulation of dosing regimens

**FIGURE 4:**
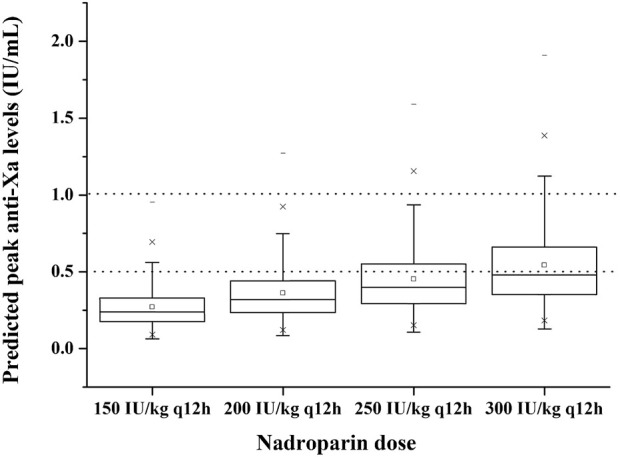
Predicted peak anti-Xa levels at 4 h with different dosing regimens to 1,000 virtual neonates and infants under 8 months. The boxplots show the minimum, first quartile, median, third quartile and maximum predicted peak anti-Xa level. The dotted lines represent the therapeutic range of anti-Xa (lower and upper bound).

Using the individual PK parameter values obtained by Bayesian forecasting with the final model, we calculated and illustrated in Figure 4 the achieved t = 4 h anti-Xa levels for the entire cohort. Our findings revealed that a dose of 150 IU/kg q12 h resulted in only 8.6% of patients falling within the desired anti-Xa level range. However, when increasing the dosing regimen to 200 IU/kg q12h, this percentage rose to 18.0%. Further escalation to a dosing regimen of 250 and 300 IU/kg q12 h led to an increased proportion of patients (28.4% and 38.5%, respectively) achieving targeted anti-Xa levels for the total cohort. Nevertheless, it is worth noting that there was an occurrence of anti-Xa levels exceeding 1 IU/mL in a small subset of patients (3.3% and 8.1%, respectively) with the dosing regimen of 250 and 300 IU/kg q12 h.

## Discussion

A population PK model was developed to characterize the relationship between dose and anti-Xa levels in neonates and infants receiving thromboprophylactic treatment with nadroparin at a dose of 150–200 IU/kg q12 h. To our knowledge, only a few previous population PK models of nadroparin have been published, including studies in morbidly obese bariatric surgery patients (Diepstraten et al., 2015), pediatric open heart surgery patients (Laporte et al., 1999), patients receiving hemodialysis (Jaspers et al., 2022), critically ill adult patients (Diepstraten et al., 2023), and COVID-19 intensive care unit patients (Piwowarczyk et al., 2023; Romano et al., 2023). However, no population PK analyses have been conducted for nadroparin in neonates and infants. This study demonstrates that the PK parameters of nadroparin may be influenced by the characteristics of this population. For neonates and infants (PNA <8 months), the clearance was estimated to be 0.068 L/h/kg. Only one study conducted by Silvy Laporte et al. investigated pediatric open heart surgery patients aged between 15 days and 8 years (no premature infants were included), reporting an estimated clearance of 0.037 L/h/kg^20^. In comparison, the clearance of nadroparin in adults is estimated at around 0.018 L/h/kg for morbidly obese and non-obese patients aged between 22 and 59 years old (Diepstraten et al., 2015) or approximately at a rate of 0.025 L/h/kg for COVID-19 intensive care unit patients aged between 53 and 70 years old (Romano et al., 2023). These findings suggest that a higher dosage is required in neonates and infants than older children and adults to reach similar anti-Xa levels.

Among all tested covariates, creatinine clearance with an exponential model for clearance yielded the largest improvement in model fit, which is consistent with nadroparin primarily undergoing renal excretion. A population pharmacokintic analysis of nadroparin for thromboprophylaxis in COVID-19 intensive care unit patients aged 53–70 years found that the clearance of nadroparin was associated with renal clearance (Romano et al., 2023). P Mismetti et al. investigated whether renal function influences the pharmacokinetic pattern and observed that anti-Xa clearance of nadroparin in elderly healthy patients (creatinine clearance: 62 ± 6 mL/min) was 1.4 times lower than in young healthy patients (creatinine clearance: 114 ± 15 mL/min) (Mismetti et al., 1998). An expert review suggested adjusting nadroparin to 50%–65% and 75%–85% of the original dose for patients with a creatinine clearance of <30 mL/min and 30–60 mL/min, respectively (Broek et al., 2022). In a review article by Nagge et al., it was concluded that renal insufficiency affects the clearance of each LMWH differently. Data from trials involving nadroparin support the notion that accumulation of anti-Xa heparin activity may occur in patients with renal impairment. The clearance of nadroparin was found to be reduced below a creatinine clearance threshold of 50 mL/min (Nagge et al., 2002). Body weight with an allometric exponent of 0.75 for clearance was also incorporated as a covariate model. However, during the backward elimination, the increase in OFV was not statistically significant (*p* > 0.01). Allometric size adjustments using a fixed coefficient of 0.75 for clearance have repeatedly been used in pediatric PK analyses. Nevertheless, careful consideration must be given to the possibility that allometric scaling may not hold true for all studied populations. Additionally, we investigated the impact of postmenstrual and postnatal age on clearance as the coagulation system develops over time, both factors demonstrated a statistically significant effect when they were individually tested. However, with the incorporation of creatinine clearance, neither postmenstrual age nor postnatal age showed significant effects. This observation may be partially attributed to high correlation between creatinine clearance and development- or maturation-related parameters such as age in pediatric patients.

The optimal dosing regimen of nadroparin in neonates and infants remains to be determined, as guidelines for the use of LMWHs in adults cannot be directly extrapolated to children, particularly neonates, due to variations in pharmacokinetics and the influence of immature hemostatic system. Previously suggested anti-Xa therapeutic window of 0.5–1 IU/mL allows estimation of the probability of achieving adequate anti-Xa levels for different dosages. In our center, only a small subset of patients treated with current dosing regimens (150–200 IU/kg) achieved anti-Xa levels within this recommended therapeutic range; however, these regimens appeared safe since no major bleedings occurred. During treatment with nadroparin, one neonate died as a result of their underlying disease, three neonates were transferred to other hospitals, and one neonate was discontinued due to abnormal blood clotting. Among the remaining 35 pediatric patients, complete clot resolution was observed in 18 cases (51.4%), partial clot resolution was observed in 13 patients (37.1%), and no improvement was observed in 4 patients (11.4%). Model simulations demonstrated that 28.4% patients reached TTR with a dosage of 250 IU/kg q12h, indicating higher doses are required to achieve TTR compared to older children. These findings are consistent with previous studies. Van Ommen’s et al. conducted a prospective study involving two preterm and ten term neonates treated with nadroparin therapy at an initial dosage of 85.5 IU/kg q12 h, eventually six of them (50%) achieved TTR between 0.5 and 1.0 IU/mL with a mean dosage of 224 ± 21 IU/kg q12 h but required significantly more time compared to older children (Ommen et al., 2008). Jeanine Sol et al. investigated sixty-one preterm and term neonates with 64 venous thromboembolisms, where fifty percent (32/64) reached TTR and the median nadroparin dosage required to reach TTR was found to be 197 (97.9–330.3) IU/kg q12 h (Sol et al., 2021).

This study is subject to certain constraints. First, it was developed using data from a limited number of patients. Given the infrequent occurrence of thromboembolism in children, limited clinical experiences exist regarding the use of nadroparin in neonates and infants. Obtaining blood samples for anti-Xa level measurements can be challenging in this vulnerable population, resulting in less frequent monitoring of anti-Xa levels. According to the local protocol, peak concentrations are typically monitored, while trough concentrations can be assessed in cases of suspected drug accumulation. However, in our center, all the samples we obtained are peak concentrations, thereby limiting a comprehensive understanding of the shape of the entire PK curve. The applicability of using this model in a clinical setting where trough concentrations are being collected remains unknown. Neonates and infants usually require higher dosages of nadroparin to achieve TTR compared to adults, however, caution should be exercised by physicians when increasing nadroparin dosages in this population due to increased bleeding risk. Consequently, a considerable proportion of patients (21.6%) exhibited concentrations below 0.1 IU/mL. According to a study conducted by Ron J. Keizer et al., for the linear one-compartment intravenous model, no systematic bias was observed for the population parameters CL and V when using the “Discard”, “LLOQ/2” and “LIKE” methods, in situations where there was a “moderate” (10%) or “high” (20%) percentage of BLOQ censoring (Keizer et al., 2015). In our study, sensitivity analysis was performed using the M3-method to investigate whether the exclusion of samples below the limit of quantification could introduce any bias into the model. Second-order conditional estimation with the Laplace method was applied for parameter estimation. The estimated typical values for CL/F, V/F and Ka were found to be 0.289 L/h, 1.3 L, 0.671 h^-1^ respectively, which did not show any significant differences compared to those estimated by the “Discard” method. These results provide evidence that employing the “Discard” method does not lead to any systematic bias in estimating population parameters such as CL, V and Ka. Additionally, there appears a bias towards lower population predicted anti-Xa concentrations. Despite thorough examination of these data points, regrettably, the source of this bias remained unidentified. It is anticipated that larger sample sizes in future studies will facilitate the identification and rectification of this bias.

## Conclusion

The PK parameters for nadroparin in preterm or term neonates and infants less than 8 months were estimated using the model developed herein for the first time. Our results may be used to recommend the initial individualized dosage in hospitals in patient populations with similar characteristics.

## Data Availability

The original contributions presented in the study are included in the article/Supplementary material, further inquiries can be directed to the corresponding authors.
